# Live-cell imaging of DNA damage and cell cycle progression uncovers distinct responses during neural differentiation of hiPSCs

**DOI:** 10.1016/j.jbc.2025.110328

**Published:** 2025-06-03

**Authors:** Mikio Shimada, Yoshihisa Matsumoto, Kensuke Otsuka

**Affiliations:** 1 Zero-Carbon Energy Laboratory, Institute of Integrated Research, Institute of Science Tokyo, Tokyo, Japan; 2 Biology and Environmental Chemistry Division, Sustainable System Research Laboratory, Central Research Institute of Electric Power Industry, Chiba, Japan

**Keywords:** DNA repair, cell cycle, live cell imaging, induced pluripotent stem cell, focicle

## Abstract

Ionizing radiation induces DNA double-strand breaks, which compromise genomic stability and trigger programmed cell death. The cell’s differentiation state modulates DNA damage response (DDR) mechanisms, including DNA repair pathways and cell cycle regulation. The accumulation of p53-binding protein 1 (53BP1) at DSB sites serves as a reliable biomarker for such damage. Previously, we developed a fluorescent live-cell imaging system, termed “Focicle,” which monitors 53BP1 foci dynamics and cell cycle phases, utilizing fluorescent ubiquitination-based cell cycle indicators (hCdt1 and hGmnn) in mouse cells. In the current study, to investigate the relationship between differentiation state and DDR activity, we generated Focicle-integrated human induced pluripotent stem cells and further differentiated them into neural progenitors and mature neurons using an optimized Focicle cassette adapted for human cell lines. Using laser microirradiation, we observed differentiation-dependent alterations in 53BP1 foci accumulation dynamics and cell cycle progression. The newly established Focicle system represents a valuable tool for elucidating DDR activity during organ development.

Ionizing radiation (IR) induces DNA double-strand breaks (DSBs), which are predominantly repaired *via* two major pathways: homologous recombination (HR) and non-homologous end joining (NHEJ). HR repair is the most active during the S/G2 phase of the cell cycle and relies on a sister chromatid as a homologous template. Conversely, conventional NHEJ (c-NHEJ) directly ligates DNA ends and operates throughout the cell cycle, particularly during the G1 phase (1). Stem cells, including tissue stem cells and pluripotent stem cells (PSCs), exhibit distinct DNA damage response (DDR) mechanisms, including DNA repair, cell cycle checkpoints, and apoptosis, at the transcriptional level. Previous research has demonstrated the elevated expression of HR-related factors (*e*.*g*., BRCA1, BRCA2, RAD51, and NBS1) and NHEJ-related factors (*e*.*g*., XRCC4, KU70, and KU80) in PSCs (2). Meanwhile, PSCs demonstrate a short G1 status and silenced G1/S checkpoint activation through the downregulation of the cyclin-dependent kinase inhibitor proteins p16, p21, p27, and p57 (3, 4, 5, 6, 7, 8, 9, 10, 11, 12, 13, 14). In PSCs, the short G1 phase causes DNA replication stress and increases genome instability (15), and an accelerated cell cycle is likely to be required for maintaining the pluripotency of PSCs. Indeed, a delayed G1/S transition induced by transient p21 expression results in cellular differentiation, indicating that a balanced cell cycle progression is critical for sustaining pluripotency in PSCs (3, 15, 16). Furthermore, high levels of apoptosis-related factors, such as Trp53, CAS3, and BID, suggest that DNA-damaged PSCs are efficiently eliminated *via* apoptosis (17). After differentiation into neural progenitor cells (NPCs), the expression levels of some proteins involved in DDR mechanisms, including those involved in cell cycle checkpoints and apoptosis, decrease, suggesting that the genome maintenance machinery is dependent on the differentiation state and tissue type (2). However, the relationship between DNA repair activity and tissue specificity remains unclear.

The fluorescent ubiquitination-based cell cycle indicator (Fucci) system is a powerful tool for visualizing the cell cycle state using live-cell imaging. Two distinct fluorescent protein-fused cell cycle indicators, hCdt1 and hGmnn, reveal G1 and G2/M phases, respectively (18, 19). hCdt1 is present in the G1 phase but not in the G2/M phase, whereas hGmnn is present in the G2/M phase but not in the G1 phase. The levels of hCdt1 and hGmnn proteins are regulated by the activities of the SKP1-CUL1-F-box-protein complex (an E3 ligase that adds ubiquitin) and the anaphase-promoting complex/cyclosome complex, which directly or indirectly inhibit each other (20, 21). In a previous report, based on the Fucci system, we added a DSB foci indicator, the p53 binding protein (53BP1) foci-forming region (FFR), termed the (DSB foci and cell cycle) Focicle system for mouse cells (22, 23). 53BP1 is a DSB repair factor and is often used as a DSB marker. 53BP1 antagonizes HR by inhibiting DNA end resection and promoting NHEJ repair. The 53BP1 protein is recruited to DSB sites by recognizing histone H2A ubiquitination at Lys15 (H2AK15u) and histone H4 dimethylation at Lys20 (H4K20me2) (24). 53BP1FFR contains a Tudor domain that recognizes H4K20me2 and is required for its recruitment to DSB sites. Thus, the Focicle system allows simultaneous detection of the DDR and cell cycle status after genotoxic stress, such as IR exposure, as well as in the steady state.

In this study, to address the relationship between DDR activity and differentiation state, we modified the Focicle system for human cells using the PiggyBac transposon system and established Focicle-integrated human induced pluripotent stem cells (hiPSCs). A small population of Focicle hiPSCs was in the G1/S phase, which is consistent with a previous report (9). After IR and laser microirradiation exposure, Focicle hiPSCs showed slow and low 53BP1 foci accumulation, whereas Focicle human NPCs (hNPCs) and Focicle human mature neurons (hNeurons) showed rapid and high accumulation of 53BP1 foci, suggesting that differentiation state influences DDR foci formation speed. Furthermore, 24 h after IR, hiPSCs showed accumulation of hCdt1-positive cells (G1 phase), which colocalized with 53BP1 nuclear body (53BP1NB) foci. Focicle hiPSC systems and our findings will contribute to clarifying tissue- and differentiation-dependent DDR alterations.

## Results

### The function of focicle reporter gene was validated in the human RPE-hTERT cell line

**Figure 1:**
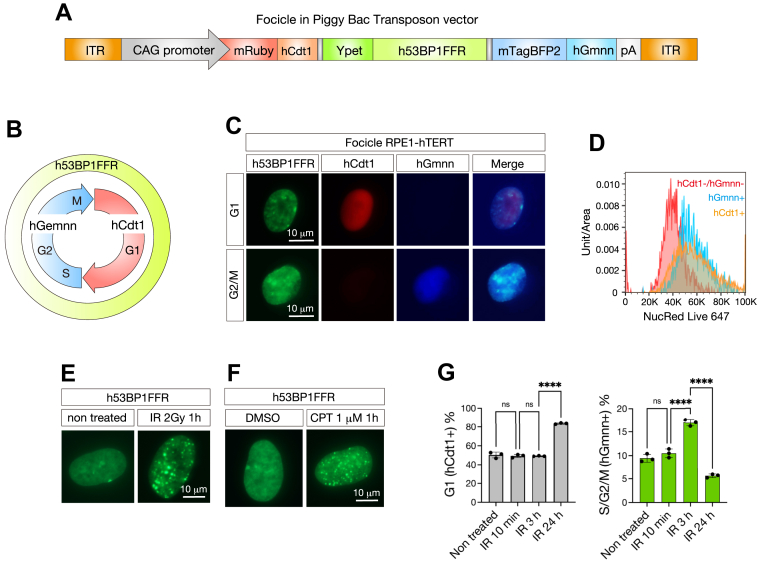
**Construction of human Focicle vector based on PiggyBac transposon system**. *A*, design of PiggyBac transposon-based human Focicle vector. Tricistronic inserts of mRuby-hCdt1, Ypet-h53BP1 foci-forming region (FFR), and mTagBFP2-hGmnn are connected to P2A and T2A peptides and expressed under the CAG promoter. *B*, scheme of 53BP1, hCdt1, and hGmnn expression in cell cycle. *C*, generation of Focicle vector-integrated human RPE1-hTERT cells. Expression of 53BP1FFR (*green*), hCdt1 (*red*), and hGmnn (*blue*) was observed using a fluorescence microscope. hCdt1 positive cells indicate cells in G1 phase (*top*). hGmnn-positive cells indicate cells in the G2/M phase (*bottom*). Scale bars represent 10 μm. *D*, the distribution of DNA content in the hCdt1^-^/hGmnn^-^ (*red*), hCdt1^+^ (*orange*), and hGmnn^+^ (*blue*) populations. *E*, Focicle RPE1-hTERT cells were exposed to 2 Gy gamma-ray, and 1 h after irradiation, 53BP1 foci were observed using fluorescence microscopy. *F*, Focicle RPE1-hTERT cells were treated with camptothecin (CPT) for 1 h, and 53BP1 foci were observed by fluorescence microscopy. *G*, cell cycle population after ionizing radiation (IR) exposure in Focicle RPE1-hTERT. Cells were untreated or treated with 2 Gy gamma-ray irradiation and analyzed at the indicated times after exposure. Cell cycle population was assessed by flow cytometry. hCdt1 positive cells were counted as G1 cells, and hGmnn-positive cells as G2 cells. Statistical analyses were performed using a one-way ANOVA and Tukey’s *post hoc* test. ns indicates no significant difference. ∗∗∗∗*p* < 0.0001. All experiments were performed at least three times.

For simultaneous detection of DSB foci and cell-cycle progression, we previously developed a murine 53BP1 foci-forming region (53BP1FFR) sequence and Fucci (hCdt1 and hGmnn)-combined fluorescent reporter system, termed “Focicle,” for targeting the mouse Rosa 26 locus (22). To analyze human cell lines, we constructed a new hFocicle vector containing human 53BP1FFR (25) and Fucci under the control of the CAG promoter in the PiggyBac plasmid (Fig. 1, *A* and *B*). To confirm whether this hFocicle system functions in human cells, we co-transfected the hFocicle vector and PiggyBac transposase expression vector into human telomerase reverse transcriptase-immortalized retinal pigment epithelial cells (RPE1-hTERT) using Lipofectamine 3000. Two days after the transfection, puromycin was added for selection. After 10 to 14 days of incubation, single-cell sorting of yellow fluorescent protein (YFP)-positive cells into a 96-well plate was performed using a cell sorter to establish cell clones and stable clones expressing Ypet-53BP1FFR, mRuby3-hCdt1 (30–120), and mTagBFP2-hGmmn (1–110) were obtained. In the obtained clones, mRuby3-hCdt1 and mTagBFP2-hGmmn were exclusively expressed during each cell cycle (Fig. 1*C*). We confirmed the DNA content using NucRed Live 647 ReadyProbe reagent and demonstrated that mRuby3-hCdt1 and mTagBFP2 were specifically expressed in the G1 and G2/M phases, respectively (Fig. 1*D*). One hour after exposure to IR through a ^60^Co source, Focicle RPE1-hTERT cells showed 53BP1 foci formation, indicating that h53BP1FFR functions in human cells (Fig. 1*E*). Furthermore, we confirmed that 53BP1 formed foci upon treatment with camptothecin, an anticancer drug that induces DNA DSBs (Fig. 1*F*). The cell cycle status after 2 Gy irradiation was analyzed using flow cytometry (Fig. 1*G*). In a steady-state (non-irradiated) control, the proportion of cells in the G1 phase (hCdt1^+^/hGmnn^-^) was 50.33 ± 3.07% (n = 3) and that in the S/G2/M phase (hCdt1^-^/hGmnn^+^) was 9.47 ± 0.82% (n = 3), which is a reasonable feature of somatic cells (26). Thus, we concluded that this new hFocicle cassette is useful for evaluating cell cycle status and DDR in human cells.

### hiPSCs showed a different DNA damage response compared to NPCs

**Figure 2:**
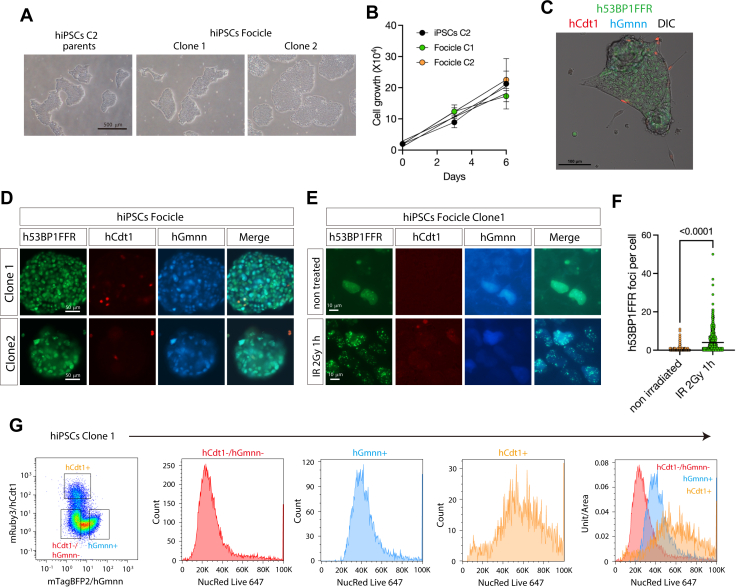
**Focicle vector-integrated human iPS cells**. *A*, generation of Focicle vector-integrated human induced pluripotent stem cells (hiPSCs). Cell morphology of Focicle hiPSCs was observed by phase contrast microscopy in two independent clones (clones one and 2) and parental cells (C2). The scale bar represents 500 μm. *B*, to assess growth rate, cell numbers were counted after seeding on the indicated days in two independent clones (clone one and clone 2) and parental cells (C2). (*C* and *D*) Generation of Focicle vector-integrated hiPSCs. The expression of 53BP1FFR (*green*), hCdt1 (*red*), hGmnn (*blue*), and differential interference contrast image (DIC) were observed by confocal microscopy (*C*) and fluorescence microscopy (*D*) in two independent clones (clone one and clone 2). Scale bars represent 100 μm (*C*) and 50 μm (*D*). (*E* and *F*) Focicle hiPSCs were either untreated or irradiated with 2 Gy. One hour after irradiation, 53BP1 foci formation were counted and graphed in F. Scale bars represent 10 μm. (*G*) Distribution of DNA content in the hCdt1^-^/hGmnn^-^ (*red*), hCdt1^+^ (*orange*), and hGmnn^+^ (*blue*) population in an hiPSC clone.

Differences in radiation sensitivity between stem cells and differentiated cells suggest distinct dynamics of cell cycle progression and DDR mechanisms across hiPSCs, hNPCs, and hNeurons. To investigate this relationship, we introduced the hFocicle cassette into hiPSCs. Using Lipofectamine Stem Reagent, we co-transfected the hFocicle vector and PiggyBac transposase expression vector into hiPSCs. Puromycin selection was applied 2 days post-transfection, and YFP-positive cells were sorted as single cells into 96-well plates after 7 to 10 days of incubation. Two stable clones (C1 and C2) expressing hFocicles were then isolated. Microscopic observations confirmed that hFocicle integration did not affect cell growth or morphology (Fig. 2, *A* and *B*). The stable expression of Ypet-53BP1FFR, mRuby3-hCdt1, and mTagBFP2-hGmmn was confirmed by fluorescence microscopy (Fig. 2, *C* and *D*). To evaluate the functionality of the hFocicle cassette in hiPSCs, we exposed the cells to 2 Gy of gamma radiation from a ^60^Co source. Following 1 h of incubation, radiation-induced 53BP1 foci formation was observed, confirming that the 53BP1FFR cassette functioned properly in hiPSCs (Fig. 2, *E* and *F*). The cell cycle status was assessed using flow cytometry. Cell nuclei were stained with NucRed Live 647 reagent. In a steady-state (non-irradiated) control, the proportion of cells in the G1 (hCdt1^+^/hGmnn^-^) phase was 4.56 ± 0.10% (C1) and 4.39 ± 0.34% (C2). The proportion of cells in S/G2/M (hCdt1^-^/hGmnn^+^) fraction was 41.0 ± 0.96% (C1, n = 3) and 34.97 ± 1.42% (C2, n = 3), a population tendency consistent with typical PSC character (Fig. 2*G*) (15, 16).

**Figure 3:**
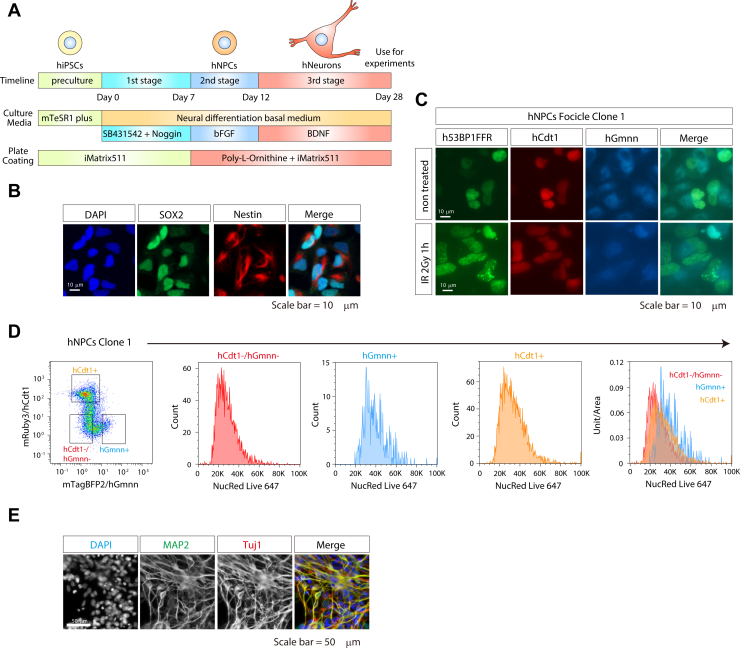
**Induction of human neural progenitor cells (hNPCs) from Focicle hiPSCs**. *A*, Scheme for neural differentiation of hiPSCs. Day 7–Day 12 cells were used as hNPCs, and after Day cells were used as neurons. *B*, hNPCs were derived from Focicle hiPSCs. To confirm neural differentiation, cells were stained for the NPCs markers SOX2 (*green*) and nestin (*red*). 4′,6-diamidino-2-phenylindole (DAPI: *blue*) was used for cell nucleus staining. The scale bar represents 10 μm. *C*, to confirm whether 53BP1FFR works in hNPCs, cells were exposed to 2 Gy gamma rays, and 53BP1 foci were observed 1 h after irradiation. Scale bars represent 10 μm. (*D*) The distribution of DNA content in the hCdt1^-^/hGmnn^-^ (*red*), hCdt1^+^ (*orange*), and hGmnn^+^ (*blue*) populations in a hNPC clone. *E*, hNeurons were derived from Focicle hiPSCs. To confirm neural differentiation, cells were stained for the mature neuron markers MAP2 (*green*) and Tuj1 (*red*). DAPI (*blue*) was used for cell nucleus staining. Scale bars represent 10 μm.

Next, to investigate the DDR in the differentiated state, we induced NPCs and neurons from hiPSCs using neuronal differentiation methods, as described previously (Fig. 3*A*) (2, 27, 28). After NPC induction, cells were assessed using NPC markers, SRY-box transcription factor 2 (SOX2), intermediate filament protein, and nestin antibodies (Fig. 3*B*). Ypet-53BP1FFR, mRuby3-hCdt1, and mTagBFP2-hGmnn expression was observed (Fig. 3*C*), and 53BP1 foci increased after radiation exposure, indicating that 53BP1FFR worked in differentiated cells. Flow cytometric analysis showed that the proportion of G1 (hCdt1^+^/hGmnn^-^) cells increased to 19.93 ± 0.31% (C1) and 29.77 ± 1.31% (C2), suggesting that differentiation prolonged the G1 phase (Fig. 3*D*). We further differentiated hNPCs into hNeurons. Neurons were confirmed with immunostaining using mature neuron markers, microtubule-associated protein 2 (MAP2) and β-tubulin III/Tuj1 antibodies (Fig. 3*E*).

### HR repair activity in hiPSCs, hNPCs, and hNeurons

**Figure 4:**
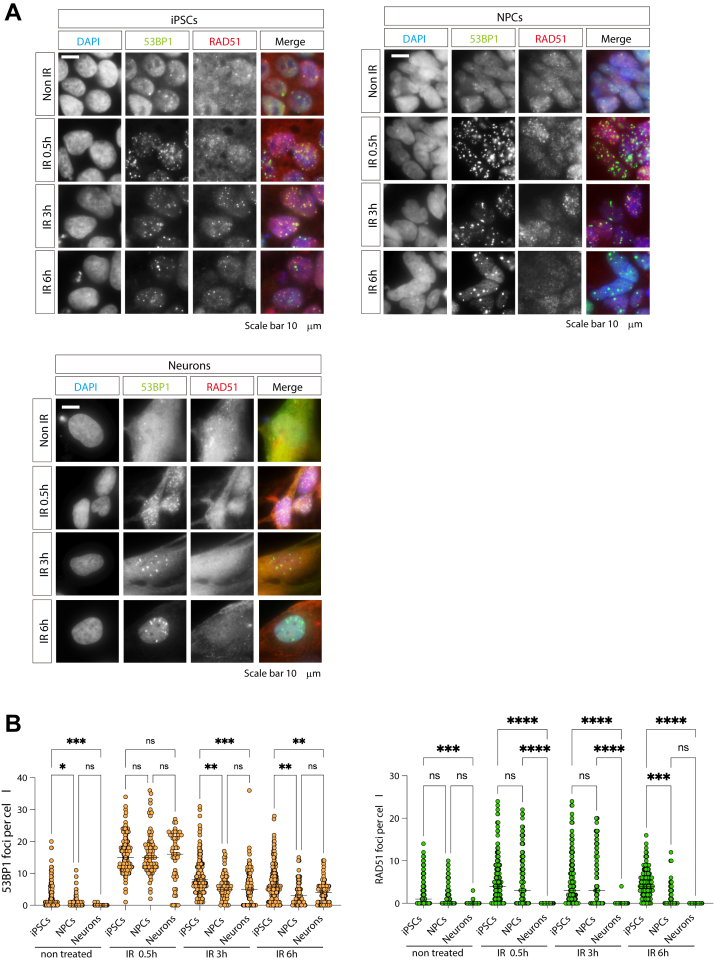
**Homologous recombination repair activity in hiPSCs, hNPCs, and hNeuro**ns. *A*, hiPSCs, hNPCs, and hNeurons were exposed to 2 Gy gamma rays and fixed at the indicated times. Non-IR cells were used as an untreated control. Cells were stained with 53BP1 and the homologous recombination marker RAD51 antibody. The cell nucleus was stained with DAPI. Scale bars represent 10 μm. *B*, quantification of 53BP1 and RAD51. The DNA damage foci per cell were quantified using CellProfiler. Statistical analyses were performed using a one-way ANOVA and Tukey’s *post hoc* test. ns indicates no significant difference. ∗*p* < 0.1, ∗∗*p* < 0.001, ∗∗∗*p* < 0.001, ∗∗∗∗*p* < 0.0001. All experiments were performed at least three times.

To evaluate the DSB repair capacity of PSCs and neural lineages, the cells were exposed to 2 Gy of irradiation, fixed at 0.5, 3, and 6 h post-exposure, and analyzed by immunostaining with various antibodies. Markers for DSBs included 53BP1 and γ-H2AX antibodies; RAD51 antibody served as an indicator of HR repair (Fig. 4*A*). Previous findings have suggested that PSCs have high HR repair activity, which decreases as they differentiate. Since mature neurons do not divide, they do not have sister chromatids that can serve as templates for HR; therefore, HR repair is significantly suppressed. In fact, the formation of RAD51 foci, which are involved in HR, was significantly reduced in hNPCs compared to hiPSCs after radiation exposure and was almost not observed in hNeurons (Fig. 4*B*), consistent with previous findings. Collectively, these results, supported by immunohistochemical analysis, confirm that RAD51-dependent HR activity is markedly diminished in hNPCs and hNeurons derived from established Focicle iPSCs.

### NHEJ repair activity in hiPSCs, hNPCs, and hNeurons

**Figure 5:**
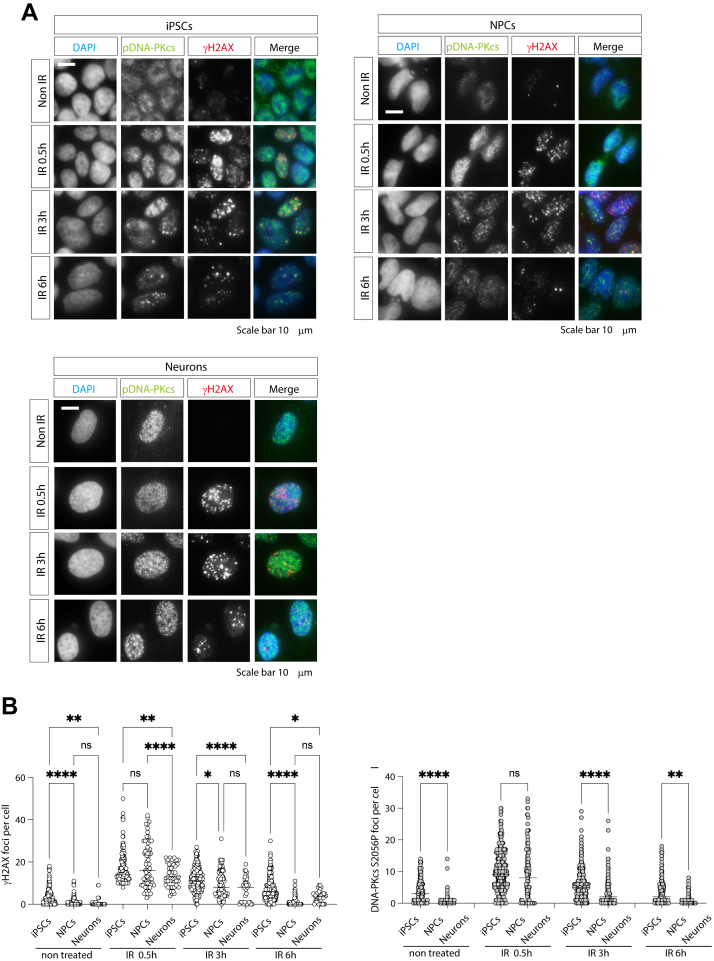
**Non-homologous end joining repair activity in hiPSCs, hNPCs, and hNeurons**. *A*, hiPSCs, hNPCs, and hNPCs were exposed to 2 Gy gamma rays and fixed at the indicated times. Non-IR cells were used as an untreated control. Cells were stained with γH2AX and non-homologous end-joining repair marker pDNA-PKcs (S2056) antibodies. The cell nucleus was stained with DAPI. Scale bars represent 10 μm. *B*, Quantification of γH2AX and pDNA-PKcs. DNA damage foci per cell were quantified using CellProfiler. Statistical analyses were performed using a one-way ANOVA Tukey’s *post hoc* test. ns indicates no significant difference. ∗*p* < 0.1, ∗∗*p* < 0.001, ∗∗∗*p* < 0.001, ∗∗∗∗*p* < 0.0001. All experiments were performed at least three times.

c-NHEJ is active throughout the cell cycle because it does not depend on templates such as sister chromatids and is thought to be the main repair pathway, especially in hNeurons and other highly differentiated cells. Therefore, we used an antibody against Ser 2056 phosphorylated DNA-protein kinase catalytic subunit (pDNA-PKcs), which is important for c-NHEJ, as a marker for c-NHEJ repair (Fig. 5, *A* and *B*). When hiPSCs and hNPCs were stained with pDNA-PKcs antibody, some foci that did not colocalize with γ-H2AX were observed, and distinguishing between nonspecific and functional foci was not possible. However, we analyzed foci that at least colocalized with γ-H2AX, assuming that they were foci involved in DSBs. In both hiPSCs and hNPCs, γ-H2AX and pDNA-PKcs foci decreased in association with γ-H2AX foci at 3 h after radiation exposure, indicating that γ-H2AX and pDNA-PKcs were linked. Furthermore, hNPCs showed a faster decrease in foci than hiPSCs did, indicating that DNA repair progressed faster in hNPCs than in hiPSCs. Conversely, in hNeurons, while the number of foci colocalized with γ-H2AX during pDNA-PKcs staining was significantly reduced, a substantial number of foci remained unassociated with γ-H2AX. This lack of colocalization complicates their identification as definitive DNA repair markers, rendering their quantification unfeasible.

### Laser microirradiation revealed protective potential against DSB foci formation of hiPSCs

**Figure 6:**
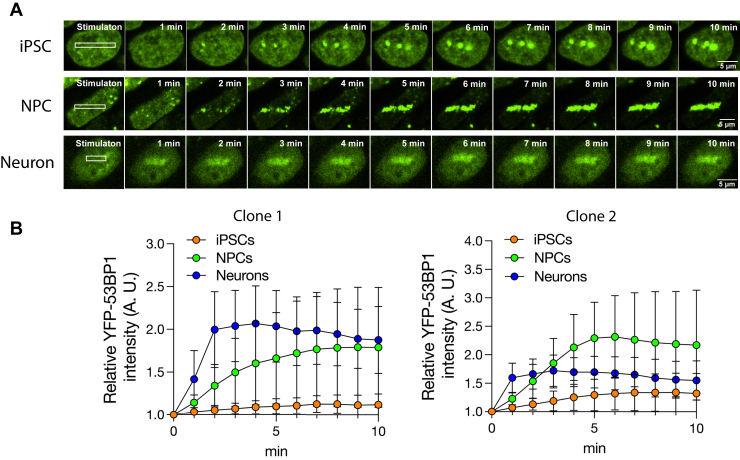
**Live cell imaging after laser microirradiation in Focicle hiPSCs, hNPCs, and hNeurons**. *A*, hiPSCs, hNPCs, and hNeurons were exposed to 405 nm laser ablation, and YFP-53BP1 foci tracks were observed at the indicated time points (minutes). Scale bars represent 5 μm. *B*, YFP-53BP1 signal intensity in Focicle hiPSCs, hNPCs, and hNeurons (two independent clones) was measured and graphed. Error bars indicate standard error. At least 30 cells were analyzed at each time point (min).

To address DNA repair dynamics, we performed live-cell imaging and laser microirradiation using confocal microscopy with focicle-integrated hiPSCs, hNPCs, and hNeurons. The shape of the cell nuclei was determined based on the fluorescence intensity of the YFP signal. We exposed a 405 nm laser inside the nuclei [1 × 10 μm region of interest (ROI)] and measured foci track formation by the ROI intensity every 1 min for up to 10 min. Although Hoechst and BrdU are commonly used sensitizers to enhance the efficiency of DSB formation, these chemicals may disrupt fluorescence. Therefore, we performed the assay in the absence of sensitizers. After microirradiation, foci tracks accumulated most rapidly in hNeurons, followed by hNPCs and hiPSCs. Although the overall accumulation varied among clones, it was higher in hNPCs and hNeurons than in undifferentiated hiPSCs (Fig. 6, *A* and *B*, Videos 1 and 2). Thus, we conclude that the recruitment of 53BP1 to sites of DNA damage was physically weaker in hiPSCs than in hNPCs and hNeurons.

### hiPSCs showed cell cycle arrest for effective DSB repair in the G1 phase after irradiation

**Figure 7:**
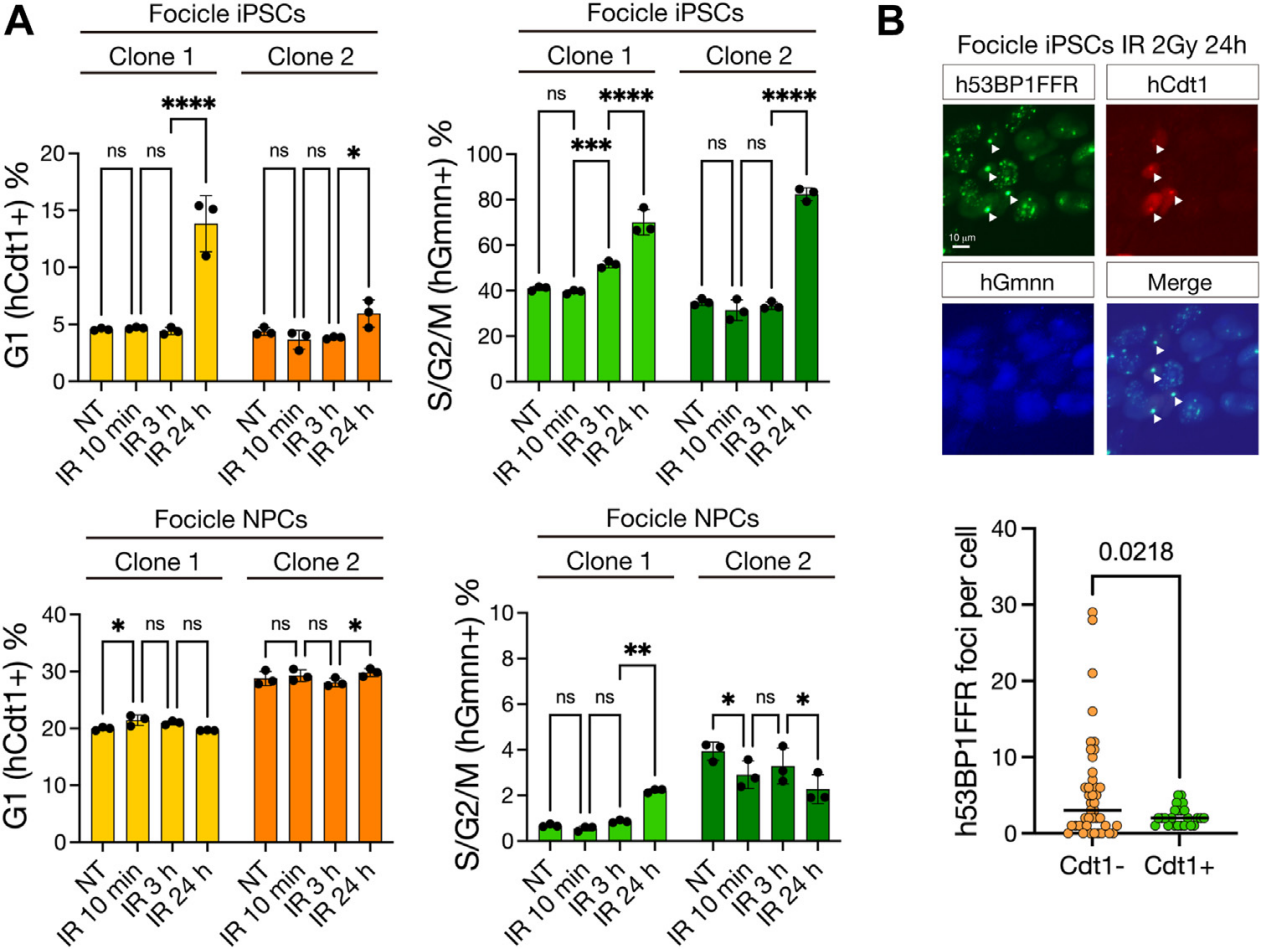
**Cell cycle analysis after IR exposure in Focicle hiPSCs and hNPCs**. *A*, cell cycle population of Focicle hiPSCs and hNPCs after IR exposure. Cells were untreated or treated with 2 Gy gamma-ray irradiation and analyzed at the indicated times after exposure. Cell cycle population was assessed by flow cytometry. hCdt1 positive cells were counted as G1 cells, and hGmnn-positive cells as G2 cells. Statistical analyses were performed using a one-way ANOVA Tukey’s *post hoc* test. ns indicates no significant difference. ∗*p* < 0.1, ∗∗*p* < 0.01. ∗∗∗*p* < 0.001, ∗∗∗∗*p* < 0.0001. All experiments were performed at least thrice. *B*, fluorescence images of h53BP1FFR, hCdt1, and hGmnn in Focicle hiPSC 24 h after IR 2 Gy exposure. Focicle hiPSCs were either not treated or irradiated with 2 Gy. Twenty-four hours after irradiation, the h53BP1FFR foci were counted and graphed. At least 100 cells were analyzed. *Arrowheads* indicate 53BP1NB foci in hCdt1 positive cells. Scale bar represents 10 μm.

To analyze the effect of exogenous genotoxic stress on the cell cycle progression of PSCs, we irradiated hFocicle-integrated hiPSCs and hNPCs with 2 Gy of gamma rays. 10 minutes after irradiation, the populations of hCdt1- and hGmnn-positive cells did not show significant changes in either hiPSCs or hNPCs. However, 24 h after irradiation, hCdt1-and hGmnn-positive cells were considerably increased in hiPSCs (G1, Clone 1: non-treated (NT): 4.56%, 24 h: 13.83%. Clone 2: NT: 4.39%, 24 h: 5.95%, S/G2/M: Clone 1: NT: 41.00%, 24 h: 70.03%. Clone 2: NT: 34.97%, 24 h: 82.40%) but not in hNPCs (G1, Clone 1: NT: 19.93%, 24 h: 19.63%. Clone 2: NT: 29.77%, 24 h: 29.80%, S/G2/M: Clone 1: NT: 0.68%, 24 h: 2.21%. Clone 2: NT: 3.94%, 24 h: 2.28%; Fig. 7*A*).

Recent reports have shown that 53BP1 forms DNA damage-associated small foci and specialized chromatin-associated large foci, which are localized to NBs (29). 53BP1NBs are important for the protection against residual DNA damage and are distinct from DNA damage-associated small foci. The number of 53BP1 small foci peaked at 1 h after IR exposure and gradually disappeared, suggesting that damaged DNA was repaired, although some cells retained 53BP1 small foci. Conversely, 53BP1NBs appeared with decreasing numbers of small foci and remained in hiPSCs for a long time. Notably, 24 h after IR exposure, 53BP1NBs preferentially localized to hCdt1-positive cells rather than to the other cells (Fig. 7*B*), suggesting that a prolonged G1 phase might be important for the protection of unrepaired DNA damage in hiPSCs. In contrast, 53BP1NBs did not appear in hNPCs. These results suggest that exogenous genotoxic stress induces a prolonged G1 phase, which is important for protection against residual DNA damage in PSCs.

## Discussion

Cell cycle-associated DNA repair machinery is crucial for genome maintenance in all tissues and organs. However, owing to technical limitations, comparing differentiation state-dependent alterations in DDR activity has been challenging. In this study, we optimized the Fucci and DNA repair (53BP1) combined reporter system, termed "Focicle," for human cells and introduced it into hiPSCs, enabling precise comparisons of DDR activity across different cell lineages. Using laser microirradiation, we revealed that the DSB foci formation activity changes during differentiation in human cells. Notably, hiPSCs showed lower foci formation than hNPCs or hNeurons did. The accumulation of 53BP1 was observed in all cell types within 1 min post-irradiation, with the highest accumulation in hNeurons, followed by hNPCs and hiPSCs. Cells at advanced stages of differentiation rapidly accumulated 53BP1. Furthermore, while 53BP1 accumulation peaked 5 min post-irradiation in hiPSCs and hNPCs, it peaked at 1 min and remained stable for up to 10 min in hNeurons. Although 53BP1 accumulation may not directly reflect overall DDR activity, it could indicate the strength of certain DDR components. The relatively low 53BP1 accumulation in hiPSCs aligned with their low DNA repair activity, as evaluated using γH2AX as a marker (Fig. 5). These findings reflect the inherent properties of hiPSCs. PSCs are expected to have high DNA repair activity, including the formation of DSB foci (30). However, a recent study revealed that the balance between efficient DNA repair and elimination of cells with damaged DNA by apoptosis is important for genome integrity in PSCs (17, 31). In a previous report, DNA repair activity was measured after radiation exposure using γH2AX foci as an indicator. PSCs were slower at DNA repair than differentiated NPCs, suggesting that the high expression of DNA repair factors in PSCs does not necessarily indicate high DNA repair activity (2). This might be attributed to the spontaneous stress of PSCs, which are constitutively placed under replication stress because of their short G1 phase (32, 33). Since PSCs show reduced fork speed and frequent fork reversal, some HR factors, such as the single-strand binding proteins RAD51 and RPA, are required for the protection of single-stranded DNA from stalled forks (15, 34). Therefore, the action of highly expressed DNA repair factors, instead of ectopic DNA damage repair, may be required to resolve DNA damage due to endogenous replication stress.

The kinetics of recruitment of DNA repair factors to DSB sites remain poorly understood. Differences in DSB generation processes and chromatin structure may underlie the variability in DNA repair capacity between hiPSCs and hNPCs. Since 53BP1 regulates heterochromatin integrity *via* liquid-liquid phase separation (34), differentiation-dependent mechanisms may contribute to variations in DNA repair factor recruitment.

Our results reveal that iPSCs responded differently from hNPCs and hNeurons, with hiPSCs exhibiting an increase in the proportion of cells in the G1 phase after DNA damage induction. In contrast, hNPCs and hNeurons displayed minimal cell cycle arrest following genotoxic stress and repaired extensive DNA damage within a short time. This finding suggests that the biological significance of DNA damage varies between undifferentiated cells, which aim to preserve their population, and differentiated cells, which have a finite functional lifespan.

The two main DSB repair pathways, HR and c-NHEJ, depend on the cell cycle status (1). Although HR is dominant in the S/G2 phase of somatic cells, c-NHEJ is active throughout the cell cycle, especially during the G1 phase. Given the extremely short G1 phase of PSCs, the role of c-NHEJ in PSCs remains unclear. As a master regulator of the DSB repair pathway, 53BP1 promotes c-NHEJ. After IR exposure, the proportion of hCdt1 (G1 phase) and hGmnn (G2 phase)-positive cells increased in hiPSCs but not in hNPCs. We observed residual 53BP1NBs in hCdt1-positive hiPSCs, suggesting that 53BP1NBs may facilitate the repair of complex DNA damage in the prolonged G1 phase through c-NHEJ. Notably, as elongation of the G1 phase induces cell differentiation, 53BP1 NB-dependent c-NHEJ may also play a role in initiating differentiation in PSCs. However, further investigation is required to confirm this hypothesis.

In summary, hiPSCs and hiPSC-derived tissue organoids are promising tools in regenerative medicine. Because PSCs are constitutively placed under replication stress due to their short G1 phase, there may be an unknown mechanism for genome maintenance. Further research on stem cell biology is essential to control the genomic stability and pluripotency of PSCs. For this purpose, our novel Focicle reporter will contribute to the understanding of the genome maintenance machinery in PSCs.

## Experimental procedures

### Cell culture

RPE1-hTERT was obtained from the American Type Culture Collection. RPE1-hTERT was maintained with Dulbecco’s modified Eagle’s medium (DMEM; Nacalai Tesque) supplemented with 10% fetal bovine serum (Hyclone, GE Healthcare) and penicillin/streptomycin (Nacalai Tesque) at 37 °C under 5% CO_2_ conditions. hiPSC C2 was derived from human neonatal skin fibroblasts NB1RGB (RIKEN BioResource Center, Japan) using the Stemgent StemRNA-NM Reprogramming Kit (Stemgent), as described previously (2). hiPSCs were seeded with iMatrix 511 silk (Nippi) and Y27632 ROCK inhibitor (Chemscene), including mTeSR plus medium (Stem Cell Technology). After 24 h of incubation, the medium was replaced with only mTeSR plus medium. All cell lines were tested for *Mycoplasma* contamination using the e-Myco *Mycoplasma* Detection PCR Kit (iNtRON Biotechnology, Inc., cat# 25235).

### Neuronal differentiation

hiPSCs were seeded in an iMatrix 511-coated six-well plate at a density of 3 × 10^5^ cells/well in mTeSR plus medium containing 10 μM Y27632 and penicillin/streptomycin. After 1 day, culture medium was changed to 200 ng/ml Noggin (149–08861, Wako) and 10 μM SB431542 (192–16541, Wako) containing neural differentiation medium, with 50% DMEM/F12 (Wako), 50% neurobasal medium (21,103,049, Thermo Fisher Scientific), 1 × N2 supplement (17,502,001, Thermo Fisher Scientific), 1 × B27 supplement (17,504,001, Thermo Fisher Scientific), 5 μg/ml insulin (093–06351; Wako), 1 mM L-glutamine (25,030,149; Thermo Fisher Scientific), 2 × nonessential amino acids (139–15651; Wako), and 100 U/ml penicillin and 100 μg/ml streptomycin (15,140,122; Thermo Fisher Scientific). The medium was changed every day. At day 7 of neuronal differentiation, cells were dissociated with TrypLE Select and seeded into six-well plates at a density of 2 × 10^6^ cells/well in 10 μM Y27632 and 20 ng/ml bFGF (060–04543; Wako) containing neuronal differentiation medium on 0.01% (wt/vol) Poly-L-ornithine- and 3.76 mg/ml iMatrix511-coated 24-well plastic plates (P3655; Sigma-Aldrich, 892,012; Nippi, 142,475; Thermo Fisher Scientific). The medium was changed every day. At Day 12 of neuronal differentiation, cells were dissociated with TrypLE Select and seeded into six-well plates at a density of 2 × 10^6^ cells/well in 10 μM Y27632 and 20 ng/ml brain-derived neurotrophic factor (BDNF; 022–16454; Wako) containing neural differentiation medium on 0.01% (wt/vol) Poly-L-ornithine- and 3.76 mg/ml iMatrix511-coated 24-well plastic plates. The medium was replaced every 3 days with 20 ng/ml BDNF-containing neural differentiation medium. On day 28 of neuronal differentiation, cells were used for immunofluorescence and live-cell imaging.

### hFocicle PiggyBac vector construction

The basic strategy for constructing the Focicle vector has been previously reported (22). For the human cell assay, the foci-forming region of human TP53BP1 (25) was amplified by conventional PCR using Platinum SuperFi DNA polymerase (Thermo Fisher Scientific, #12351–050) using specific primers for seamless cloning (forward primer: AGCTGAACGGATCCCAGGGAGAAGAAGAGT, reverse primer: GCCAAGCTTGCATGCCGAGCTCTCATTCACCGGTGTTGTC). The PCR products were purified using conventional agarose gel electrophoresis following column-based purification. To construct the PiggyBac vector, we assembled three inserts (mRuby3/hCdt1, YPet/h53BP1FFR, and mTagBFP2/hGmnn) into a PiggyBac backbone vector, which contained a puromycin resistance gene as a selection marker (Vectorbuilder), using the GeneArt Seamless PLUS Cloning and Assembly Kit (Thermo Fisher Scientific, #A14603). The ligated vectors were transfected into DH10 B T1SA competent cells.

### Establishment of focal-integrated cells

Cells were co-transfected with the hFocicle PiggyBac vector and transposase expression vector. For transfection, Lipofectamine 3000 (Invitrogen, Thermo Fisher Scientific) was used for RPE1-hTERT, and Lipofectamine Stem Transfection Reagent (Invitrogen, Thermo Fisher Scientific) was used for iPSCs. Transfection was performed according to the manufacturer’s instructions. Two days after transfection, 0.8 μg/ml puromycin was added to fresh medium for positive selection. After 10 to 14 days of incubation in puromycin-containing medium, YFP-positive cells were sorted as single-cell clones into a 96-well plate using FACS Melody (BD Biosciences). Expression of YFP/h53BP1FFR, mRuby3/hCdt1, and mTagBFP2/hGmnn was confirmed by fluorescence microscopy.

### Time-lapse live-cell imaging and laser microirradiation

A confocal microscope (A1R HD25; Nikon) equipped with a stage-top incubation chamber (TOKAI Hit) was used. For live-cell imaging, we cultured RPE1-hTERT in 35 mm glass bottom dishes (Matsunami Glass Ind., Ltd) and hiPSCs, hNPCs, or hNeurons in 35 mm microslide dishes (μ-slide, ibidi) and set the dish in a stage-top incubator at 37 °C, 5% CO_2_ condition. Fluorescent proteins were excited using 405, 488, and 561 nm lasers. The fluorescence signals from blue fluorescent protein (BFP), YFP, and red fluorescent protein (RFP) were detected with a GaAsP detector using a 405/488/561/640 nm dichroic mirror and 450/50, 525/50, and 595/50 bandpass filter cubes, respectively. Fluorescent images were captured using NIS-Elements AR (version 5.21.00) software. For laser microirradiation, we also used NIS-Elements AR software and defined the parameters for stimulation based on a report by Levone *et al*. (35). A Plan Apo λ 60 × oil immersion objective lens (numerical aperture 1.4) was used for imaging with immersion oil. Foci formation was observed by 405 nm laser ablation without sensitization using BrdU or Hoechst. Laser power was set to 100%, and the line averaging/integration count was set to 4. ROIs (1 μm height, 10 μm width) were defined as the part of the irradiated area in the cell nucleus. These ROIs were scanned using a 405 nm laser at a rate of 4 s/frame. Immediately after the scan was completed, the system waited for 10 s and then acquired fluorescence images every minute for 10 min. The background ROI was set at a different location from that of the cell. The YFP fluorescence intensity was measured in the area containing the laser-irradiated ROI, and the time course of the fluorescence intensity was examined.

### Flow cytometry

Cell cycle analysis of hFocicle-integrated cells was performed using the MoFlo Astrios EQ Flow Cytometry System (Beckman Coulter, Inc.). To compare the expression distribution of cell-cycle indicator genes with the amount of nucleic acids, NucRed Live 647 ReadyProbes Reagent (#R37106, Thermo Fisher Scientific) was added to the culture medium at the appropriate amount, according to the manufacturer’s instructions. The cells were collected 30 min after the addition. The fluorescence of BFP, YFP, RFP, and NucRed 647 was detected using 405, 488, 561, and 640 nm lasers, respectively. Scattergram with FCS format was obtained by Summit Software (ver. 6.3.1, Beckman Coulter, Inc.) and analyzed using the FlowJo Software (ver. 10.6.2, Becton, Dickinson & Company).

### Immunofluorescence

The cells were seeded on an 8-well micro slide chamber (ibidi). The cells were fixed with 4% paraformaldehyde for 10 min at room temperature and permeabilized with 0.5% Triton X-100/phosphate-buffered saline with Tween 20 (PBS-T) for 5 min at 4 °C. The cells were blocked with 1% BSA/PBS-T at 4 °C for 1 h and stained with primary antibodies: SOX2 rabbit polyclonal antibody (1:500, Human Neural Stem Cell Immunocytochemistry Kit, A24354, Invitrogen, Thermo Fisher Scientific), Nestin mouse monoclonal antibody (1:500, Human Neural Stem Cell Immunocytochemistry Kit, A24354, Invitrogen, Thermo Fisher Scientific), MAP2 chicken polyclonal antibody (1:500, #ab5392, Abcam), Tublin β3 (Tuj1) mouse monoclonal antibody (#MMS-435P, Covance), 53BP1 rabbit polyclonal antibody (1:1000, #A300–272A; Bethyl), S139P γH2AX mouse monoclonal antibody (1:1000, #05–636; Merck Millipore), RAD51 mouse polyclonal antibody (1:500, #H00005888-B01P; Abnova), phosphor S2056 DNA-PKcs rabbit polyclonal antibody (1:500, #ab18192, Abcam) for 4 h–24 h at 4 °C. After washing with PBS-T, the cells were subsequently incubated with the following secondary antibodies: Alexa-488-conjugated anti-rabbit immunoglobulin IgG, 1:1000 (Thermo Fisher Scientific), Alexa-555-conjugated anti-mouse IgG, 1:1000 (Thermo Fisher Scientific), and 4′,6-diamidino-2-phenylindole (DAPI). After washing with PBS-T, the cells were incubated in PBS. Fluorescence was visualized using an Axio Observer fluorescence microscope (Zeiss).

### Irradiation

Cells were X-ray-irradiated at 2 Gy with an MBR-1520R-4 system (Hitachi Ltd) operated at 150 kV, with a 20-mA tube current and a 0.5-mm Al + 0.3-mm Cu filter. The dose rate was 0.6 Gy/min. For gamma-ray irradiation, cells were irradiated using a ^60^Co source at the Chiyoda Technol Cobalt Center, Institute of Science, Tokyo, Japan. The dose rate was measured using an ionizing chamber-type exposure dosimeter C-110 (Oyo Giken) and corrected for decay.

### 53BP1 foci measurement and statistical analysis

The 53BP1 foci were measured using the CellProfiler tool developed at the Broad Institute of MIT and Harvard (36). All experiments were performed at least three times. Statistical analysis was performed using Welch’s (one-tailed) *t* test and one-way analysis of variance (ANOVA) followed by Tukey’s *post hoc* test. Microsoft Excel and GraphPad Prism 10 were used for statistical analysis.

## Data availability

The data that support the findings of this study are available upon reasonable request from the corresponding author.

## Supporting information

This article contains supporting information.

## Conflict of interest

The authors declare that they have no conflicts of interest with the contents of this article.
