# Supplementary material for: Live-cell imaging of DNA damage and cell cycle progression uncovers distinct responses during neural differentiation of hiPSCs
Source: J Biol Chem. 2025 Jun 3;301(7):110328. doi: 10.1016/j.jbc.2025.110328 (PMC12268688; doi:10.1016/j.jbc.2025.110328)
Supplement: Supplementary File [file mmc3.docx]

**Supporting information**

This article contains supporting information below.

Supplemental Video 1: Live cell imaging after laser microirradiation in Focicle hiPSCs

Supplemental Video 2: Live cell imaging after laser microirradiation in Focicle hNPCs
